# A phase 1 study of lenvatinib, multiple receptor tyrosine kinase inhibitor, in Japanese patients with advanced solid tumors

**DOI:** 10.1007/s00280-015-2899-0

**Published:** 2015-11-03

**Authors:** Shinji Nakamichi, Hiroshi Nokihara, Noboru Yamamoto, Yasuhide Yamada, Kazunori Honda, Yosuke Tamura, Hiroshi Wakui, Tatsuya Sasaki, Wataru Yusa, Katsuki Fujino, Tomohide Tamura

**Affiliations:** Department of Thoracic Oncology, National Cancer Center Hospital, Tokyo, Japan; Department of Gastrointestinal Oncology, National Cancer Center Hospital, Tokyo, Japan; Clinical Development, Eisai Co., Ltd., Tokyo, Japan; Biostatistics, Eisai Co., Ltd., Tokyo, Japan; St. Luke’s International Hospital, Akashi-cho 9-1, Chuo-ku, Tokyo, 104-8560 Japan

**Keywords:** Lenvatinib, E7080, Phase 1, Angiogenesis, Japanese, Safety

## Abstract

**Purpose:**

This phase 1 study aimed to assess the tolerability, safety, pharmacokinetics (PK), pharmacodynamics, and preliminary efficacy of lenvatinib capsules in Japanese patients with solid tumors when administered orally up to 24 mg on a once-daily (QD) continuous schedule.

**Methods:**

Patients were enrolled in one of the two sequential cohorts (20 or 24 mg) of lenvatinib on a 28-day cycle based on the conventional 3 + 3 dose escalation design. Adverse events (AEs) were graded using the Common Terminology Criteria for Adverse Events, version 4.0. Tolerability was judged based on dose-limiting toxicities (DLTs) during Cycle 1. The drug was defined as tolerable when the incidence of DLTs was less than 33 %.

**Results:**

Nine patients received lenvatinib [20 mg (*n* = 3); 24 mg (*n* = 6)]. No DLTs were observed. The most common AEs were thrombocytopenia, blood thyroid stimulating hormone increased, and hypertension (89 %), followed by leukopenia, headache, and proteinuria (78 %). The area under the concentration–time curve and maximum observed concentration increased dose proportionally. The PK profiles were similar to those in non-Japanese phase 1 studies. One patient with leiomyosarcoma showed a partial response, and three patients have maintained stable disease for more than 6 months.

**Conclusions:**

The 24-mg QD continuous dose of lenvatinib was determined to be tolerable with encouraging anti-tumor activity in Japanese patients with solid tumors.

## Introduction

Angiogenesis, the formation of new blood vessels from a preexisting vascular network, is essential for tumor growth and metastasis [1, 2]. Vascular endothelial growth factor (VEGF) receptors 1–3 are expressed on endothelial cells and play essential roles in both physiologic and pathologic angiogenesis [3]. Lenvatinib is an oral inhibitor of multiple receptor tyrosine kinases targeting VEGF receptors 1–3, fibroblast growth factor (FGF) receptors 1–4, platelet-derived growth factor receptor α, ret proto-oncogene, and v-kit Hardy-Zuckerman 4 feline sarcoma viral oncogene homolog (c-Kit) [4, 5]. In preclinical models, lenvatinib inhibits VEGF- and FGF-driven proliferation and tube formation of human umbilical vein endothelial cells in vitro [6]. In vivo angiogenesis induced by overexpressed VEGF or FGF was significantly suppressed with oral lenvatinib treatments [6].

Phase 1 studies of lenvatinib have been conducted in patients with solid tumors exploring a wide range of dose levels and with different dosing schedules: once-daily (QD) continuous dosing in the European Union (EU) [7], twice-daily (BID) continuous dosing in the USA [8], and BID intermittent dosing (2 weeks on/1 week off) in Japan [9]. Based on clinical and pharmacokinetic (PK) data from these phase 1 studies, the recommended dose of lenvatinib as a single agent has been determined to be 24-mg continuous QD dosing. Lenvatinib has shown encouraging anti-tumor activity in phase 2 studies in patients with thyroid cancer [10], endometrial cancer [11], renal cancer [12], and non-squamous, non-small cell lung cancer [13]. Recently, lenvatinib demonstrated a significant improvement in progression-free survival compared with placebo in patients with radioiodine-refractory differentiated thyroid cancer in a phase 3 study (SELECT study) and has recently been approved for this indication [14]. The toxicities in this study were manageable with dose modification and medical therapy. The most common adverse events (AEs) of any grade were hypertension, diarrhoea, fatigue or asthenia, decreased appetite, decreased weight, and nausea.

The previous phase 1 studies used a tablet formulation, although the final formulation for the phase 3 study was a capsule. A clinical pharmacology study with healthy volunteers showed that dose modification is not necessary between the lenvatinib tablet and capsule formulation [15]. However, no phase 1 study of lenvatinib capsules in solid tumor patients has been reported. Furthermore, no PK or safety data for lenvatinib in Japanese patients with solid tumors have been reported when administered QD continuously. Therefore, we conducted a dose escalation phase 1 study of lenvatinib capsules up to 24 mg QD continuously in Japanese patients with advanced solid tumors.

## Materials and methods

### Patients

Eligible patients were aged 20 years or older with a cytologically or histologically documented solid tumor that was refractory to standard therapy, or for which no standard therapy was available. Eligible patients also had completed anticancer therapy more than 4 weeks prior to lenvatinib treatment, and their toxicities had recovered to Grade 1 or lower except for alopecia. Additional inclusion criteria were Eastern Cooperative Oncology Group performance status (ECOG PS) of 0–1, adequate bone marrow function (hemoglobin ≥9.0 g/dL, neutrophil count ≥1.5 × 10^3^/μL, platelet count ≥10 × 10^4^/μL), liver function (total bilirubin ≤1.8 mg/dL, aspartate aminotransferase [AST] ≤100 IU/L, alanine aminotransferase [ALT] ≤100 IU/L), and renal function (creatinine ≤1.5 mg/dL or creatinine clearance ≥50 mL/min). Patients were excluded from the study if they had brain metastasis that was symptomatic or required treatment, complication or history of interstitial pneumonia, complication of pulmonary fibrosis with clinical symptoms, systemic infections requiring treatment, major cardiovascular diseases (ischemic cardiac disease or arrhythmia, angina pectoris or myocardial infarction within 24 weeks prior to enrollment, or QTc greater than 480 ms), hemoptysis, hemorrhagic or thrombotic events within 4 weeks prior to enrollment, hypertension (systolic blood pressure ≥150 mm Hg or diastolic blood pressure ≥90 mm Hg), or proteinuria (≥2+ in a qualitative test for urine protein or if ≥1+ proteinuria remained 2 days or more at ≥1.0 g for 24 h accumulated), history of surgery that would influence absorption of the investigational drug, major surgery within 4 weeks prior to enrollment, and coexisting effusion requiring treatment. Patients were also excluded if they were unable to take oral lenvatinib, were being treated with drugs that strongly inhibit or induce CYP3A4, or were positive for human immunodeficiency virus or hepatitis B or C virus.

### Study design

This was a single-center, open-label, dose escalation phase 1 study to assess the tolerability, safety, PK, pharmacodynamics (PD), and preliminary efficacy of lenvatinib administered orally up to 24 mg QD on a continuous schedule in patients with solid tumors (clinicaltrials.gov identifier NCT01268293). This study was conducted in accordance with the Declaration of Helsinki and was approved by the institutional review board at the participating institution. Written informed consent was obtained from all patients before any study-related procedures were performed. The study used a conventional 3 + 3 dose escalation scheme. Patients were treated in sequential cohorts of escalating doses of lenvatinib administered in 28-day treatment cycles until disease progression, development of unacceptable toxicity, or withdrawal of consent. Planned lenvatinib doses were 20 and 24 mg. The 24-mg dose was the highest planned dose because prior phase 1 studies determined the recommended dose to be 24 mg QD. If no dose-limiting toxicities (DLTs) were observed in three patients or if one DLT was observed in six patients at the 20-mg dose level, patient enrollment could be initiated for the 24-mg dose level. If zero or one DLT was observed in six patients at the 24-mg dose level, the dose was defined as tolerable. QD oral dose of lenvatinib was administered each morning regardless of food intake except for the day of blood sampling for PK analysis (Day 1 and Day 15 of Cycle 1) at which time lenvatinib was administered in a fasting condition. Planned doses for dose reduction were 20, 14, and 10 mg because 4 and 10 mg capsules were used. When a further dose reduction lower than 10 mg was necessary, the principal investigator and Eisai discussed the next dose on a case-by-case basis. If a DLT occurred, lenvatinib was interrupted until recovered to Grade 0–1 or baseline and then restarted at a reduced dose. All subjects who demonstrated a DLT or completed Cycle 1 could continue treatment after additional written informed consent for continuation in the study was obtained.

### Safety

Safety assessments measured from baseline through 30 days after the last dose included AE profile, laboratory variables [hematology, chemistry, urinalysis, thyroid-stimulating hormone, free triiodothyronine (T3), free thyroxine (T4), human chorionic gonadotropin], vital signs (systolic and diastolic blood pressure, pulse rate, and body temperature), weight, 12-lead electrocardiograms, ECOG PS, and physical examination. Toxicity was graded using the National Cancer Institute (Washington, DC, USA) Common Terminology Criteria for Adverse Events, version 4.0. All AEs emerging during the study were classified by standardized medical terminology using the Medical Dictionary for Regulatory Activities (MedDRA, version 15.1) and appropriately tabulated.

### DLTs

DLTs were assessed during the first treatment cycle and were defined as Grade 4 neutropenia that persists for more than 7 days, Grade ≥3 febrile neutropenia with ≥38.5 °C and neutrophils <1.0 × 10^3^/μL, Grade 4 thrombocytopenia or Grade 3 thrombocytopenia that requires blood transfusion, any Grade ≥3 non-hematologic toxicity (except for controlled hypertension, diarrhea/vomiting/nausea controlled by supportive care, Grade ≥3 ALT, AST, γ-glutamyltransferase [γ-GTP], or alkaline phosphatase [ALP] increase that persists for ≤7 days, or other Grade ≥3 abnormal clinical laboratory values that persist for ≤3 days), or any toxicity that necessitates the interruption of lenvatinib for >7 days.

### PK

Serial blood samples were collected at predose, 1, 2, 4, 8, and 24 h after the first dose on Day 1 of Cycle 1 and after repeated doses on Day 15 of Cycle 1. Blood samples at predose on Day 8 of Cycle 1 and Day 15 of Cycle 2 were also collected. Validated liquid chromatography/mass spectrometry was used to determine lenvatinib in plasma.

### PD

Blood samples for PD markers were collected on Days 1, 8, and 15. Circulating endothelial cells (CECs) and circulating endothelial progenitor cells (CEPs), which reflect active vascular turnover and angiogenesis, were measured as described previously [9]. Plasma samples were analyzed in triplicate for baseline and post-treatment levels of 10 angiogenic proteins and cytokines using BioPlex PRO™ human group I cytokine 6-plex (VEGF, platelet-derived growth factor-BB, interleukin [IL]-6, IL-8, IL-10, and granulocyte colony-stimulating factor) and group II 3-plex (stem cell factor, stromal cell-derived factor-1α, and hepatocyte growth factor [HGF]) panel assays (Bio-Rad Laboratories Inc.) and the Invitrogen™ FGF-basic singleplex bead kit (Life Technologies) by LSI Medience Corporation.

### Efficacy

Efficacy was assessed using Response Evaluation Criteria in Solid Tumors version 1.1, and assessments were conducted every 8 weeks or sooner if clinically indicated. Tumor response was defined as complete response (CR), partial response (PR), and stable disease (SD) defined as ≥7 weeks after the start of treatment or progressive disease. Disease control rate was defined as the percentage of patients with a best overall response (BOR) of CR, PR, or SD.

## Results

### Patient characteristics

Nine patients (three patients in the 20-mg dose level and six patients in the 24-mg dose level) were enrolled at the National Cancer Center Hospital, Tokyo, Japan. Baseline characteristics are summarized in Table 1. The median age was 41 years (range 30–59), and seven patients were female. All patients had ECOG PS score of 0 at baseline. Eight patients had undergone two or more prior chemotherapy regimens before enrollment in this study. Six patients had a primary diagnosis of leiomyosarcoma, one had endometrial stromal sarcoma, one had colorectal cancer, and one had melanoma. The median duration of treatment with lenvatinib was 56 days (range 28–172) in the 20-mg dose level and 209 days (range 59–520) in the 24-mg dose level. The median percentage of the received dose of lenvatinib versus the planned dose was 97 % (range 77–100) in the 20-mg dose level and 82 % (range 73–100) in the 24-mg dose level. Seven patients discontinued lenvatinib treatment due to progression of disease, one patient due to an AE, and one patient due to patient choice.

**Table 1:** Patient characteristics

Category	20 mg (*n* = 3)	24 mg (*n* = 6)
Age, years		
Median (range)	38 (32–59)	44 (30–59)
Sex, *n* (%)		
Male	0	2 (33)
Female	3 (100)	4 (67)
Weight (kg)		
Median (range)	48 (44–48)	60 (53–66)
ECOG performance status, *n* (%)		
0	3 (100)	6 (100)
Type of primary tumor, *n* (%)		
Leiomyosarcoma	2 (67)	4 (67)
Endometrial stromal sarcoma	1 (33)	0
Colorectal	0	1 (17)
Melanoma	0	1 (17)
Subjects with any previous anticancer surgical therapy, *n* (%)	3 (100)	6 (100)
Subjects with any previous radiotherapy, *n* (%)	1 (33)	1 (17)
Number of previous anticancer regimens, *n* (%)		
0	1 (33)	0
1	0	0
≥2	2 (67)	6 (100)

### Safety and tolerability

No DLTs were reported in this study, and both the 20- and 24-mg dose levels were judged to be tolerable. The common AEs (≥30 % of patients in the total group) reported during the study are summarized in Table 2. The most frequently observed AEs were thrombocytopenia, blood thyroid stimulating hormone increased, and hypertension (*n* = 8, 89 %), followed by leukopenia, headache, and proteinuria (*n* = 7, 78 %). There were no Grade 4 AEs. The observed Grade 3 AEs were neutropenia (*n* = 3, 33 %), blood cholesterol increased (*n* = 2, 22 %), weight decreased, diarrhoea, hypertension, hypertriglyceridemia, leukopenia, anemia, lymphopenia, and proteinuria (*n* = 1, 11 %). No patient died or had an AE that resulted in death on treatment or within 30 days of the last dose. One serious AE, Grade 3 diarrhoea occurred in one patient in the 24-mg dose level and was considered possibly related to the study drug by the investigator. The patient recovered from this with supportive care and dose interruption. AEs leading to study drug withdrawal occurred in one patient (11 %) (Grade 1 arthralgia and Grade 2 palmar-plantar erythrodysesthesia [PPE] syndrome) in the 24-mg dose level. AEs leading to study drug dose reduction occurred in two patients (22 %): one patient with Grade 3 proteinuria in the 20-mg dose level and one patient with Grade 2 stomatitis and Grade 2 oropharyngeal pain in the 24-mg dose level. AEs leading to study drug interruption occurred in six patients (67 %): one patient in the 20-mg dose level and five patients in the 24-mg dose level. The most frequently occurring AEs leading to study drug interruption included proteinuria (*n* = 3, 33 %), followed by hypothyroidism, abdominal pain upper, malaise, hypoalbuminemia, and edema peripheral (*n* = 2, 22 %).

**Table 2:** Adverse events (any grades) with an overall incidence ≥30 %

MedDRA preferred term	20 mg (*n* = 3) *n* (%)		24 mg (*n* = 6) *n* (%)		Total (*n* = 9) *n* (%)	
	Any G	G3	Any G	G3	Any G	G3
Any term	3 (100)	3 (100)	6 (100)	3 (50)	9 (100)	6 (67)
Thrombocytopenia	2 (67)	0	6 (100)	0	8 (89)	0
Hypertension	2 (67)	0	6 (100)	1 (17)	8 (89)	1 (11)
Blood thyroid stimulating hormone increased	3 (100)	0	5 (83)	0	8 (89)	0
Leukopenia	2 (67)	1 (33)	5 (83)	0	7 (78)	1 (11)
Headache	3 (100)	0	4 (67)	0	7 (78)	0
Proteinuria	2 (67)	1 (33)	5 (83)	0	7 (78)	1 (11)
Aspartate aminotransferase increased	3 (100)	0	3 (50)	0	6 (67)	0
Blood cholesterol increased	2 (67)	0	4 (67)	2 (33)	6 (67)	2 (22)
Nausea	2 (67)	0	4 (67)	0	6 (67)	0
Palmar-plantar erythrodysesthesia syndrome	2 (67)	0	4 (67)	0	6 (67)	0
Malaise	2 (67)	0	4 (67)	0	6 (67)	0
Hypertriglyceridemia	1 (33)	0	5 (83)	1 (17)	6 (67)	1 (11)
Diarrhea	0	0	5 (83)	1 (17)	5 (56)	1 (11)
Dysphonia	1 (33)	0	4 (67)	0	5 (56)	0
Arthralgia	1 (33)	0	4 (67)	0	5 (56)	0
Myalgia	0	0	5 (83)	0	5 (56)	0
Neutropenia	2 (67)	2 (67)	3 (50)	1 (17)	5 (56)	3 (33)
Alanine aminotransferase increased	3 (100)	0	2 (33)	0	5 (56)	0
Blood lactate dehydrogenase increased	2 (67)	0	2 (33)	0	4 (44)	0
Electrocardiogram T wave inversion	0	0	4 (67)	0	4 (44)	0
Abdominal discomfort	1 (33)	0	3 (50)	0	4 (44)	0
Rash	1 (33)	0	3 (50)	0	4 (44)	0
Edema peripheral	2 (67)	0	2 (33)	0	4 (44)	0
Decreased appetite	0	0	4 (67)	0	4 (44)	0
Hypoalbuminemia	2 (67)	0	2 (33)	0	4 (44)	0
Oropharyngeal pain	1 (33)	0	3 (50)	0	4 (44)	0
Blood alkaline phosphatase increased	0	0	3 (50)	0	3 (33)	0
Abdominal pain upper	0	0	3 (50)	0	3 (33)	0
Vomiting	2 (67)	0	1 (17)	0	3 (33)	0
Hematuria	3 (100)	0	0	0	3 (33)	0
Hyperthyroidism	1 (33)	0	2 (33)	0	3 (33)	0
Hypothyroidism	0	0	3 (50)	0	3 (33)	0

### PK

**Fig. 1:**
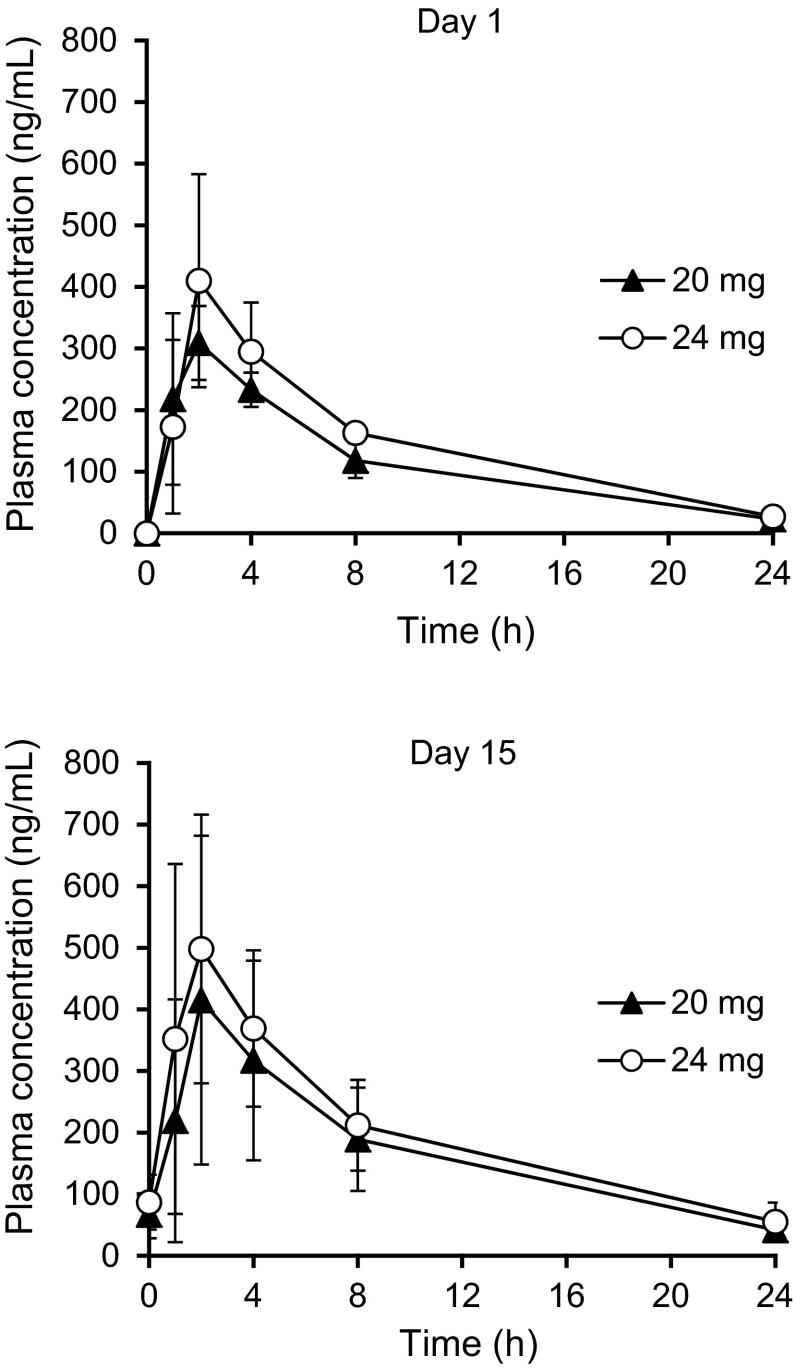
Plasma concentration versus time profile of lenvatinib following a single dose (Cycle 1, Day 1) and multiple doses (Cycle 1, Day 15)

PK parameters of lenvatinib on Day 1 and Day 15 of Cycle 1 are summarized in Table 3, and the mean plasma concentration versus time profiles are shown in Fig. 1. Following oral administration, lenvatinib was absorbed rapidly, with a maximum concentration reached within 2 h.

**Table 3:** Pharmacokinetic parameters of lenvatinib following a single dose (Cycle 1, Day 1) and multiple doses (Cycle 1, Day 15)

	20 mg (*n* = 3)	24 mg (*n* = 6)
Cycle 1, Day 1		
*C*_max_ (ng/mL)	309 ± 60.1	418 ± 167
*t*_max_ (h)	2.0 (2.0, 2.0)	2.0 (2.0, 4.0)
AUC_(0–24 h)_ (ng h/mL)	2500 ± 647	3150 ± 352^a^
Cycle 1, Day 15		
*C*_ss,max_ (ng/mL)	415 ± 267	518 ± 209
*t*_ss,max_ (h)	2.0 (2.0, 2.1)	2.0 (2.0, 4.0)
AUC_(0-τ)_ (ng h/mL)	3690 ± 1790	4140 ± 1350^b^
CL_ss_/*F* (L/h)	6.17 ± 2.34	6.19 ± 1.53^b^
*R*_ac_ (*C*_max_)	1.27 ± 0.562	1.42 ± 0.708
*R*_ac_ (AUC)	1.44 ± 0.356	1.32 ± 0.417^c^

AUC_(0–24h)_, area under the concentration–time curve from zero time to 24 h; AUC_(0-τ)_, area under the concentration–time curve over the dosing interval on multiple dosing; *C*_max_, maximum observed concentration; *C*_ss,max_, maximum observed concentration at steady state; CL_ss_/*F*, oral clearance at steady state; *t*_max_, time at which the highest drug concentration occurs; *R*_ac_, accumulation index; *t*_ss,max_, time at which the highest drug concentration occurs at steady state; *t*_1/2_, terminal elimination phase half-life

*R*_ac_ (*C*_max_) = *C*_ss,max_/*C*_max_, *R*_ac_ (AUC) = AUC_(0-τ)_/AUC_(0–24h)_

Data are the mean ± standard deviation except *t*_max_ and *t*_ss,max_; for *t*_max_ and *t*_ss,max_, median (minimum–maximum) is shown

^a^ *n* = 4

^b^ *n* = 5

^c^ *n* = 4

**Fig. 2:**
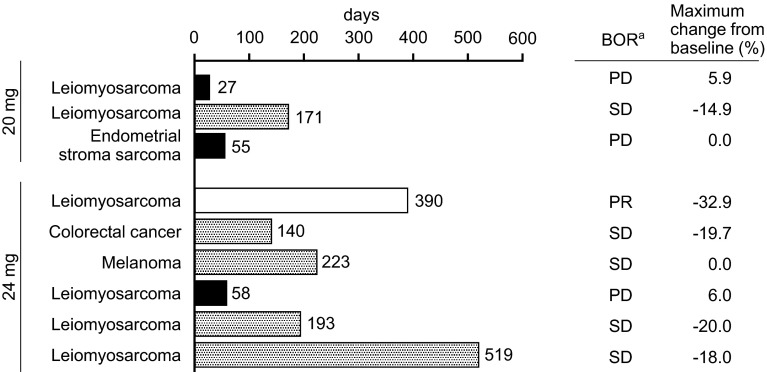
Treatment duration and anti-tumor effect of lenvatinib. ^a^Best overall response

The mean exposure, as measured by maximum observed concentration (*C*_max_) and area under the concentration–time curve (AUC), increased with an increasing dose of lenvatinib. The mean accumulation index (*R*_ac_) of *C*_max_ and AUC ranged from 1.27 to 1.42 and 1.32 to 1.44, respectively. Plasma trough concentrations of lenvatinib on Day 8, Day 15 of Cycle 1, and Day 15 of Cycle 2 remained at almost the same level, indicating that a steady state was attained with ~8 days of QD dosing (Fig. 2).

### PD

**Fig. 3:**
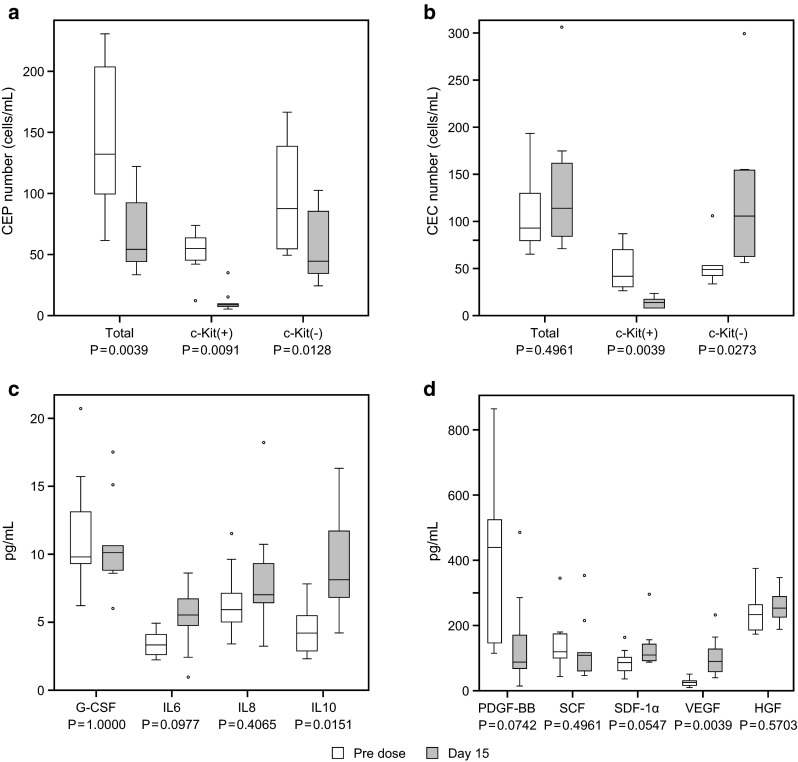
Change in the number of CEPs (**a**), CECs (**b**), and the levels of biomarkers (**c** and **d**) from predose baseline to Day 15. *P* values were calculated using the Wilcoxon signed-rank test

At 15 days after lenvatinib treatment, the total number of CEPs was significantly decreased compared with baseline levels (*P* < 0.01); this was attributable to a significant decrease in the number of c-Kit (+) CEPs (*P* < 0.01; Fig. 3a). The total number of CECs remained unchanged (*P* > 0.05), but the number of c-Kit (+) CECs was decreased significantly (*P* < 0.01; Fig. 3b). Among plasma angiogenic factors and cytokines, VEGF increased significantly (*P* < 0.01) on Day 15 compared with baseline levels (Fig. 3c, d).

### Efficacy

The BOR, duration of treatment, and maximum tumor shrinkage from baseline (%) for each patient are shown in Fig. 2. One patient with leiomyosarcoma in the 24-mg QD group had a BOR of PR with treatment duration of 390 days. This gave the overall response rate of 11 %. Five patients had a BOR of SD. Of these, four patients had durable (≥23 weeks) SD: three patients with leiomyosarcoma and one patient with melanoma. The disease control rate (CR + PR + SD) was 67 %. Tumor shrinkage from baseline was observed in five patients: one patient in the 20-mg dose level and four patients in the 24-mg dose level.

## Discussion

This dose escalation phase 1 study showed that lenvatinib at 24 mg QD had a favorable safety profile for Japanese patients with solid tumors. Based on the absence of DLTs, the 24-mg dose of lenvatinib was determined to be tolerable and recommended for further clinical studies in Japanese patients with solid tumors.

The safety profile was similar to that of typical anti-angiogenic agents. In particular, hypertension and proteinuria were common AEs. The safety profile of lenvatinib in Japanese patients with solid tumors was similar to that observed in a non-Japanese phase 1 study [7, 8]. Hypertension was well managed by antihypertensive agents without serious AE, and proteinuria was managed by dose interruption and reduction. In this study, thrombocytopenia was reported as the most frequently observed AE. In the previous phase 1 study in Japan, DLTs were reported in two patients at the 20-mg BID dose levels, both of whom experienced a Grade 3 platelet count decrease [9]. This may be due to the high exposure of lenvatinib (40 mg/day), because Grade 3 thrombocytopenia was not observed in this study. However, platelets should be carefully monitored in clinical practice in case of severe thrombocytopenia. Although PPE syndromes in this study were generally mild with no Grade 3, one patient discontinued the study drug due to Grade 2 PPE syndrome. Three patients showed Grade 2 PPE in this study; the onset of Grade 2 PPE in these patients was 50, 71, and 281 days after initiating lenvatinib treatment, suggesting that a certain period of treatment with lenvatinib can cause Grade 2 PPE. Education about early signs and symptoms of PPE for patients may be important to prevent and manage PPE syndromes.

PK parameters, including *C*_max_ and AUC, increased in a dose-dependent manner. Mean maximum concentration at steady state (*C*_ss,max_) and area under the concentration–time curve over the dosing interval on multiple dosing (AUC_(0-τ)_) after multiple doses in the 24-mg dose level by capsule in this study were 518 ng/ml and 4140 ng h/mL, respectively. In the previous phase 1 study in the EU, 24 patients received a 25-mg QD dose of lenvatinib by tablet, and the median *C*_ss,max_ and AUC_(0-τ)_ after multiple doses were 545 ng/ml and 4220 ng h/mL. A clinical pharmacology study was conducted to evaluate the effect of formulation on the PK of lenvatinib, and this study showed that the capsule formulation produces slightly lower exposure (~10 to 14 %) to lenvatinib compared with tablet formulation [15]. These data demonstrated that the PK profile in Japanese patients is similar to that observed in the EU study.

The subpopulation of CECs and CEPs may be predictive of disease or clinical responsiveness to anti-VEGF agents [18]. Lenvatinib reduced the number of CEPs but not CECs. Specifically, c-Kit (+) CEPs were reduced much more than c-Kit (−) CEPs, suggesting that Kit kinase inhibition by lenvatinib contributes to the total CEP reduction. Changes in the levels of plasma proteins may reflect the biologic response of host tissues to therapy and may be useful markers for the clinical activity of anti-tumor agents. The inhibitory effect of lenvatinib on VEGF signaling has been evaluated in preclinical studies; VEGF-induced growth of human endothelial cells is suppressed by lenvatinib [18]. The increasing VEGF level in this study suggested that lenvatinib inhibited the VEGF signaling pathway. No correlation between the clinical outcome and biomarkers in this study can be assessed due to the small number of patients.

Although efficacy was not a primary objective, encouraging preliminary evidence of anti-tumor activity was observed in Japanese patients with solid tumors. Tumor shrinkage from baseline was observed in five patients, ranging from −14.9 to −37.1 %. Interestingly, tumor shrinkage was observed in four patients with leiomyosarcoma, including one patient achieving PR. A large number of drugs have failed to provide PR or tumor shrinkage in patients with sarcoma [16]. Even pazopanib, a recently approved molecular target agent in patients with sarcoma, showed the best overall response of PR in only 6 % of patients in a phase 3 study [17]. This fact suggests that lenvatinib could be an effective drug that induces tumor shrinkage in patients with solid tumors including leiomyosarcomas. This study also indicated that lenvatinib could be administered for a long term. The median duration of treatment with lenvatinib was 209 days in the 24-mg dose level, and two patients achieved treatment duration of more than 1 year. This may be due to the effective disease control and manageable safety profile with dose modification and medical therapy.

In conclusion, lenvatinib was tolerated up to a dose of 24 mg QD in Japanese patients with solid tumors in this study. The safety and PK profiles of lenvatinib were similar to those observed in non-Japanese populations. AEs in this study were generally manageable with dose modification and medical therapy. Encouraging anti-tumor activity of lenvatinib supports further development of lenvatinib for the treatment of patients with a wide range of solid tumors.
